# Phylogenetic analysis and genotyping of Iranian infectious haematopoietic necrosis virus (IHNV) of rainbow trout (*Oncorhynchus mykiss*) based on the glycoprotein gene

**DOI:** 10.1002/vms3.931

**Published:** 2022-09-09

**Authors:** Zahra Ziafati Kafi, Arash Ghalyanchilangeroudi, Donya Nikaein, Amin Marandi, Hooman Rahmati‐Holasoo, Naser Sadri, Ahmad Erfanmanesh, Ala Enayati

**Affiliations:** ^1^ Faculty of Veterinary Medicine Department of Microbiology and Immunology University of Tehran Tehran Iran; ^2^ Faculty of Veterinary Medicine Department of Aquatic Animal Health University of Tehran Tehran Iran; ^3^ Centre of Excellence for Warm Water Fish Health and Disease Shahid Chamran University of Ahvaz Ahvaz Iran; ^4^ Academic Center for Education, Culture and Research (ACECR) Tehran Organization Tehran Iran

**Keywords:** glycoprotein gene, infectious haematopoietic necrosis, Iranian virus strain, phylogenetic analysis, rainbow trout

## Abstract

**Background:**

Infectious haematopoietic necrosis (IHN) is known as one of the most contagious systemic viral diseases in salmonids which can lead to significant mortality rates and negative impacts on the salmonid farming industry. Infectious haematopoietic necrosis virus (IHNV) was first detected in rainbow trout (*Oncorhynchus mykiss*) farms in Iran in 2003.

**Objectives:**

We conducted the present study to determine the detection of IHN genotypes in rainbow trout (*O. mykiss*) in farms in the central parts of Iran, using molecular and phylogenetic techniques.

**Methods:**

Samples were collected from fries exhibiting clinical signs such as darkening of the skin, abdominal swelling, and loss of appetite. Phylogenetic analysis was performed by the neighbour‐joining method, using MEGA 5.1 software. For phylogenetic analysis and genotyping of IHNV from central parts of Iran, the sequences of the glycoprotein gene were determined for two Iranian isolates (Jahad‐UT1 and Jahad‐UT2).

**Results:**

Phylogenetic analysis revealed that the detected strains (Jahad‐UT1 and Jahad‐UT2 isolates) are closely related (97.23%–100%) to European isolates within genogroup ‘E’.

**Conclusions:**

This finding indicates that Jahad‐UT1 and Jahad‐UT2 isolates have been widely transferred to Iran from European countries. Moreover, the nucleotide diversity of these Iranian isolates showed a close relationship with the North American and Asian isolates, although the Iranian isolates were collected from a smaller geographical area and within a shorter time period between 2014 and 2015.

## Graphical abstract

Phylogenetic analysis revealed that the detected strains (Jahad‐UT1 and Jahad‐UT2 isolates) are closely related (97.23%‐100%) to European isolates within genogroup ‘E’. This finding indicates that Jahad‐UT1 and Jahad‐UT2 isolates have been widely transferred to Iran from European countries. Moreover, the nucleotide diversity of these Iranian isolates showed a close relationship with the North American and Asian isolates.

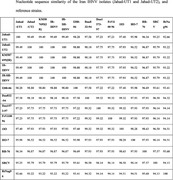

## 1 INTRODUCTION

Aquaculture of food fish is considered a major sector of the aquaculture industry (De silva, 2012; Rahmati‐Holasoo et al., 2022b; Subasinghe et al., 2009). Iran is one of the most important producers of freshwater rainbow trout (Ahmadivand et al., 2017). However, over the past decade, Iranian trout production has declined significantly due to outbreaks of some viral diseases, including VHS, IPN, and IHN (Akhlaghi & Hosseini, 2007; Ahmadivand et al., 2017, 2016, 2020b). In addition, some viral fish diseases have already been reported in Iran. Carp pox in koi (*Cyprinus carpio* L.), koi herpes virus (KHV) in koi and common carp (*Cyprinus carpio* L.), carp oedema virus (CEV) in common carp, viral haemorrhagic septicemia virus (VHSV), infectious pancreatic necrosis virus (IPNV) and infectious haematopoietic necrosis virus (IHNV) in farmed rainbow trout (*Oncorhynchus mykiss*) and lymphocystis disease in flowerhorn fish (hybrid cichlid) and Indian glassy fish (*Parambassis ranga*) have been reported (Ahmadivand et al., 2017, 2016, 2020a, 2020b; Akhlaghi & Hosseini, 2007; Rahmati‐Holasoo et al., 2010, 2016, 2022a, 2020; Ziafati Kafi et al., 2022).

Infectious haematopoietic necrosis (IHN) is one of the most contagious systemic viral diseases in salmonids, which can lead to significant mortality rates and negative impacts on the salmonid farming industry (Amar, Kiron, Akutsu, Satoh, and Watanabe, 2012; Bootland and Leong, 2011; OIE, 2019; Ahmadivand et al., 2017). Infectious haematopoietic necrosis virus (IHNV), the known causative agent of the disease, is a single‐stranded negative‐sense RNA virus belonging to the genus Novirhabdovirus of the family Rhabdoviridae (Wolf, 1988; OIE, 2019). This viral disease is usually associated with a range of clinical signs, including skin darkening, lethargy, exophthalmia, pale gills, distended abdomen and petechial haemorrhages leading to mass mortalities. However, mortalities may also occur in the absence of significant clinical signs (Purcell et al., 2013). The natural host range of IHNV includes many species of wild and farmed salmon and trout (Kurath et al., 2016). In the early 1950s, infectious haematopoietic necrosis virus was detected primarily in sockeye salmon (*Oncorhynchus nerka*) hatcheries in western North America (Rucker, Whipple, Parvin, and Evans, 1953). At that time, the virus spread to Asian and European countries via the transport of contaminated fish or eggs (Bovo, Giorgetti, Jørgensen, and Olesen, 1987; Rudakova, Kurath, and Bochkova, 2007). In Europe, the virus was reported in France and Italy in 1987 and later, in Germany in 1992(Enzmann, Kurath, Fichtner, and Bergmann, 2005).

The complete genome structure and phylogenetic relationship of infectious haematopoietic necrosis virus were first determined in 1995. IHNV possessed only one serotype, which is divided into five major genogroups: ‘U’, ‘M’, ‘L’, ‘E’ and ‘J’ (He, Ding, He, Yan, and Teng, 2013; Brieuc, Purcell, Palmer, and Naish, 2015; Purcell et al., 2013). Diseased fish exhibit bleeding of skin lesions, exophthalmia, abdominal distention, pale gills, skin darkening and extensive necrosis of haematopoietic tissues such as liver, pancreas, and granular cells of the lamina propria of the intestine (Amend and Smith, 1975). An effective method to prevent IHNV is the vaccination of healthy fish, while there are no definitive treatment protocols (Wang et al., 2016). This study aimed to examine the sequence of Jahad‐UT1 and Jahad‐UT2 strains of IHNV and then to perform a comparative sequence analysis and a phylogenetic tree analysis between these strains and other reference strains available in GenBank.

## 2 MATERIALS AND METHODS

### 2.1 Fish source and sampling

**FIGURE 1:**
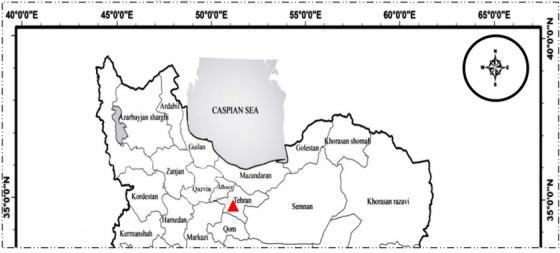
Sampling geographic locations of rainbow trout (*Oncorhynchus mykiss*) farms (▲) in central part of Iran (Tehran province in September 2014 to December 2015)

During the period from September 2014 to December 2015, an outbreak with mass mortality (70%–90%) occurred in rainbow trout (*Oncorhynchus mykiss*) farms in Tehran, Iran province (Figure 1). The farms were operated with a flow‐through system for fresh water at a temperature between 10°C and 12°C. Five moribund fish were selected from each farm and transferred to the virology laboratory. Samples were collected from fries suspected of having IHN and exhibiting clinical signs such as darkening of the skin, abdominal swelling and loss of appetite. The samples were stored at −70°C for further analysis. Pools of 10 whole fry samples were also used for molecular detection.

### 2.2 RNA extraction and cDNA synthesis

RNA was extracted from tissue samples using the CinnaPure RNA Extraction Kit (SinaClon, Iran). For cDNA synthesis, 1 μl (0.2 μg) of random hexamer primer (SinaClon, Iran) was added to 5 μl of extracted RNA, and the mixture was heated at 65°C for 5 min. 14 μl of cDNA master mix containing 7.25 μl of DEPC‐treated water (SinaClon, Iran), 2 μl of dNTP mix (SinaClon, Iran), 0.25 μl of RiboLock RNase inhibitor (Thermo Fisher Scientific, USA), 0.5 μl of Revert Aid Reverse Transcriptase (Thermo Fisher Scientific, USA) and 4 μl of 5X RT reaction buffer were added to each tube, resulting in a final volume of 20 μl. The mixture was then incubated at 25°C for 5 min, 42°C for 60 min, 95°C for 5 min and 4°C for 1 min. The cDNA was stored at −20°C until use.

### 2.3 RT‐PCR and sequencing

The reverse transcription polymerase chain reaction (RT‐PCR) for detecting IHNV was based on the OIE‐recommended primers. The forward primer is 5′‐AGA‐GATCCC‐TAC‐ACC‐AGA‐GAC‐3′ and the reverse primer is 5′‐GGT‐GGT‐GTT‐GTT‐TCC‐GTGCAA‐3′ (OIE, 2019). The reverse transcription polymerase chain reaction kit (RT‐PCR) was used according to the manufacturer's protocol (SinaClon, Iran). The thermal RT‐PCR steps included cycling at 50°C for 30 min and 95°C for 2 min, followed by 35 cycles of denaturation at 95°C for 30 s, annealing at 50°C for 30 s, and extension at 72°C for 1 min in this study. The PCR product was analysed by electrophoresis on a 1.5% agarose gel and visualised under UV light. Subsequently, the PCR products were analysed to determine the nucleotide sequences.

### 2.4 Phylogenetic analysis

Phylogenetic analysis was performed by the neighbour‐joining method, using MEGA 5.1 software, and each tree was constructed with a consensus of 1000 bootstrap repeats (Tamura et al., 2011). The nucleotide sequences of the glycoprotein gene were compared with various glycoprotein gene sequences from GenBank, including I208‐06, Dau832‐94, Dau1573‐97, FsVi100 96, 1IO, HO‐7, RB‐76, SRCV and RtNag96 (Table 1). The glycoprotein gene sequences of IHNV were deposited in the NCBI GenBank database under accession numbers KX901448.1 for isolate Jahad‐UT1 and KX901449.1 for Jahad‐UT2.

**TABLE 1:** Nucleotide sequence similarity of the Iran IHNV isolates (Jahad‐UT1 and Jahad‐UT2), and reference strains

	Jahad‐UT1	Jahad‐UT2	KM587695(IR)	IR‐IHNV	IR‐SH‐IHNV	I208‐06	Dau832‐94	Dau1573‐97	FsVi100 96	1IO	HO‐7	RB‐76	SRCV	RtNag96
Jahad‐UT1	100	99.49	99.49	99.49	99.49	98.28	97.58	97.23	97.23	97.40	95.98	96.34	95.25	92.66
Jahad‐UT2	99.49	100	100	100	100	98.80	98.10	97.75	97.75	97.93	96.52	96.87	95.79	93.22
KM587695(IR)	99.49	100	100	100	100	98.80	98.10	97.75	97.75	97.93	96.52	96.87	95.79	93.22
IR‐IHNV	99.49	100	100	100	100	98.80	98.10	97.75	97.75	97.93	96.52	96.87	95.79	93.22
IR‐SH‐IHNV	99.49	100	100	100	100	98.80	98.10	97.75	97.75	97.93	96.52	96.87	95.79	93.22
I208‐06	98.28	98.80	98.80	98.80	98.80	100	97.58	97.22	97.22	97.40	95.98	96.69	95.61	93.41
Dau832‐94	97.58	98.10	98.10	98.10	98.10	97.58	100	99.32	99.32	99.32	97.93	97.93	96.50	94.32
Dau1573‐97	97.23	97.75	97.75	97.75	97.75	97.22	99.32	100	99.32	99.32	97.93	97.93	96.14	94.14
FsVi100 96	97.23	97.75	97.75	97.75	97.75	97.22	99.32	99.32	100	99.32	97.93	97.93	96.14	94.14
1IO	97.40	97.93	97.93	97.93	97.93	97.40	99.32	99.32	99.32	100	98.28	98.63	96.50	94.51
HO‐7	95.98	96.52	96.52	96.52	96.52	95.98	97.93	97.93	97.93	98.28	100	97.93	96.14	94.51
RB‐76	96.34	96.87	96.87	96.87	96.87	96.69	97.93	97.93	97.93	98.63	97.93	100	97.57	95.60
SRCV	95.25	95.79	95.79	95.79	95.79	95.61	96.50	96.14	96.14	96.50	96.14	97.57	100	94.13
RtNag96	92.66	93.22	93.22	93.22	93.22	93.41	94.32	94.14	94.14	94.51	94.51	95.60	94.13	100

## 3 RESULTS

**FIGURE 2:**
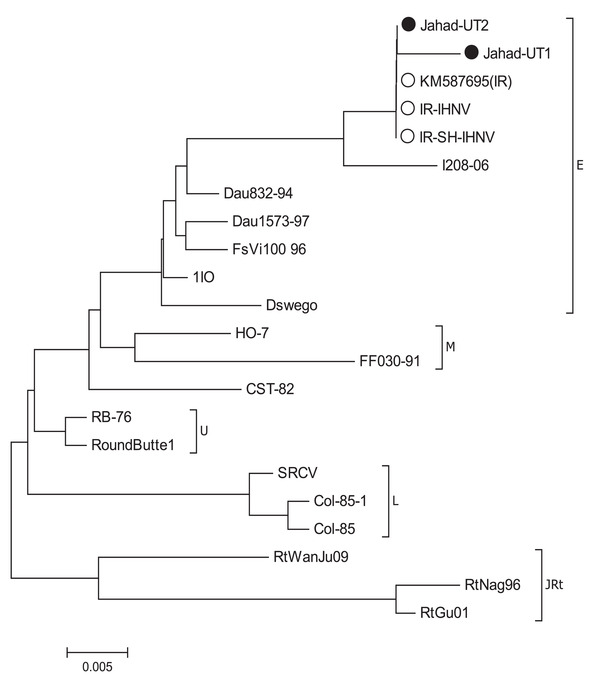
Phylogenetic tree of 2 Iranian isolates and 12 reference strains of infectious haematopoietic necrosis virus based on the nucleotide sequences of the glycoprotein gene. The phylogenetic tree was constructed, using MEGA version 5 by the neighbour‐joining method with 1000 bootstrap replicates (bootstrap values are shown on the tree). The isolates from this study are indicated by black circles. White dots indicate Iranian reference strains

Out of 10 pooled samples tested, 2 kidney tissues showed positive results in RT‐PCR (20%). Both were sent for sequencing, and their sequences were submitted to GenBank under accession numbers KX901448.1 and KX901449.1. Glycoprotein gene comparisons revealed that the viruses detected in this study belonged to genogroup ‘E’ (Figure 2). In addition, two strains from this study had the highest nucleotide similarity with IHNV detected in Iran in 2014 (Table 1).

## 4 DISCUSSION

Infectious haematopoietic necrosis caused by Infectious haematopoietic necrosis virus IHNV is an acute, highly contagious viral disease of wild and cultured salmonid fish (Bootland and Leong, 2011; Xu et al., 2019) that should be reported to the World Organization for Animal Health (OIE, 2019).

Although IHNV‐like virus was first detected by cytopathic effect (CPE), electron microscopy and IDFAT and ELISA reactions in Iran in 2003 (Fallahi et al., 2003), the first isolation of IHNV performed by reverse transcription polymerase chain reaction (RT‐PCR), was reported in Iran in 2010 (Raissy, Momtaz, Ansari, and Moumeni, 2010). Moreover, there are few molecular diagnostic studies (infection rate and genotyping) of the virus in wild farmed salmonids in Iran. We conducted the present study to determine the detection of IHN genotypes in rainbow trout (*Oncorhynchus mykiss*) in farms in the central parts of Iran, using molecular and phylogenetic techniques. Phylogenetic analysis showed that the detected strains (Jahad‐UT1 and Jahad‐UT2 isolates) are closely related (97.23%–100%) to European isolates of genogroup ‘E’. This result indicates that the Jahad‐UT1 and Jahad‐UT2 isolates were widely transferred to Iran from European countries. The results obtained in the current study are consistent with the findings of Adel et al. (2016).

The nucleotide diversity of the two Iranian isolates (Jahad‐UT1 and Jahad‐UT2) showed a close relationship with the North American and Asian isolates, although the Iranian isolates were collected from a smaller geographic area and in a more limited time period between 2014 and 2015.

The results of the current study revealed similarities with the findings of Adel et al. (2016), who found two IHN genotypes in the Iranian provinces of Mazandaran and Chaharmahal‐Bakhtiari. Moreover, Adel et al. (2016) investigated that Iranian isolates are mainly related to European isolates within the genogroup ‘E’ and not to those of North American genogroups ‘U’, ‘M’ and ‘L’, and Asian genogroup ‘J’. It appears that Iranian IHNVs were most likely introduced to Iran from a source in Europe through the transport of contaminated fish eggs (Adel et al., 2016).

In all cases where IHNV has been detected in rainbow trout (*Oncorhynchus mykiss*) in Iran, genetic typing has shown that the strains belong to the ‘E’ genogroup. Therefore, the transmission of infected fish eggs from Europe to Iran could be a possible source.

In the study conducted by Adel et al. (2016), using cell cultures and RT‐PCR an IHN prevalence of 25% was found in samples from hatcheries in Mazandaran and Chaharmahal‐Bakhtiari provinces of Iran, Raissy et al. (2010) and Adel et al. (2016) also reported an IHN prevalence of 33.3% in trout hatcheries in Chaharmahal‐Bakhtiari province Iran.

IHNV has become a significant cause of infection and mortality in farmed rainbow trout in North America, Europe and Asia following its accidental introduction via infected eggs or juveniles.

The endemic genogroups have low levels of host‐specific virulence due to genetic alterations. Kurath et al. (2016) compared the virulence of eleven strains of infectious haematopoietic necrosis virus, representing genogroups ‘U’, ‘M’ and ‘L’, in experimental challenges of juvenile Atlantic salmon in freshwater. Results showed that mortality patterns exhibited two different distinct in terms of disease progression kinetics and final per cent mortality, with nine strains exhibiting moderate virulence and two strains (genogroups ‘U’ and ‘L’) exhibiting high virulence (Kurath et al., 2016).

The global spread of novirhabdoviruses caused by aquaculture activities, notably the trade‐in eyed eggs, has been proven by the genotyping of virus strains (Bovo et al., 2021; Kurath and Winton, 2008; Winton, 1991). With an annual demand for 300–400 million eyed eggs, of which 70% are imported from European nations, Iran is one of the world's top producers of freshwater rainbow trout (Ahmadivand et al., 2017, 2016; FAO, 2020). However, some of these imports are probably not under sufficient veterinary care because of the economic and political situation. Additionally, Iranian fish farms are closely connected and exchange eggs, fry and fish annually, creating several opportunities for the virus to spread throughout the industry (Ahmadivand, Palić, and Weidmann, 2021). Therefore, the results of the current study highlight the need to implement strict regulations on the trade of fish eggs to prevent the spread of the virus to other regions of the world where rainbow trout (*Oncorhynchus mykiss*) are farmed.

## AUTHOR CONTRIBUTIONS

All authors contributed to the study conception and design. Material preparation, data collection and analysis were performed by Zahra Ziafati Kafi, Arash Ghalyanchilangeroudi, Donya Nikaein, Naser Sadri, Ahmad Erfanmanesh and Ala Enayati. The first draft of the manuscript was written by Amin Marandi and edited by Hooman Rahmati‐Holasoo, and all authors commented on previous versions of the manuscript. All authors read and approved the final manuscript.

## CONFLICT OF INTEREST

The authors have no conflicts of interest to declare that are relevant to the content of this article.

## FUNDING

The authors did not receive support from any organisation for the submitted work.

## ETHICAL STATEMENT

All procedures of the study were conducted according to a protocol approved by the ethics committee of the faculty of sciences at University of Tehran (357; 8 November 2000).

### PEER REVIEW

The peer review history for this article is available at https://publons.com/publon/10.1002/vms3.931.

## Data Availability

Research data are not shared.
